# Supplementary material for: Developmental changes in exploration resemble stochastic optimization
Source: Nat Hum Behav. 2023 Aug 17;7(11):1955–67. doi: 10.1038/s41562-023-01662-1 (PMC10663152; doi:10.1038/s41562-023-01662-1)
Supplement: Supplementary file 1 — Supplementary Figs. 1–10, Tables 1–8, Statistics, Comparability Across Experiments, Behavioural Results and Model Results. [file 41562_2023_1662_MOESM1_ESM.pdf]

nature human behaviour

Article https://doi.org/10.1038/s41562-023-01662-1

Developmental changes in exploration resemble stochastic optimization

In the format provided by the authors and unedited

nature human behaviour

Article https://doi.org/10.1038/s41562-023-01662-1

Developmental changes in exploration resemble stochastic optimization

In the format provided by the authors and unedited

Contents

1 Comparability Across Experiments 1

2 Statistics 2

2.1 Comparisons . . . 2

2.2 Correlations . . . 2

3 Supplementary Behavioral results 3

3.1 Learning over rounds regression . . . 3

3.2 Search distance regression . . . 5

4 Supplementary Model Results 6

4.1 Parameter recovery . . . 9

4.2 Comparison of Parameter Regression Models . . . 11

4.3 Lesioned Parameter Regression Models . . . 11

4.4 Parameter Regression Models . . . 12

References 18

1 Comparability Across Experiments

To assess whether the data from the different experiments are equivalent, we compared participants' performance (Supplementary Fig. 1a), the mean predictive accuracy of the GP-UCB model (Supplementary Fig. 1b), and parameter estimates for all parameters of the GP-UCB model (Supplementary Fig. 1c). Both, frequentist statistics and Bayes factors (BF) are reported (see Statistics). We compared data from the different experiments for overlapping age ranges: participants between 7-9 from Experiment 1 ($n = 36$) and Experiment 2 ($n = 22$) and participants older than 20 years from Experiment 1 ($n = 24$) and Experiment 3 ($n = 43$). Participants younger than or equal to 20 years were excluded from the comparisons, because of expected developmental changes over the course of adolescence.

Supplementary Figure 1a shows participants' performance separated by experiments. We found no difference in performance for both age groups (children between 7-9: $t(56) = 1.5$, $p = .128$, $d = 0.4$, $BF = .73$; adults older than 20: $t(65) = -0.7$, $p = .460$, $d = 0.2$, $BF = .33$). Additionally, a comparison of the predictive accuracy showed no differences in model performance between different experiments (children between 7-9: $t(56) = 0.7$, $p = .467$, $d = 0.2$, $BF = .34$; adults older than 20: $t(65) = -0.9$, $p = .394$, $d = 0.2$, $BF = .35$; see Supplementary Figure 1b). Furthermore, we compared parameter estimates for overlapping age ranges. Since parameter estimates are bounded above 0, we performed rank-based tests (Mann–Whitney U) to look for differences in parameter estimates across different experiments. The results are reported in Supplementary Table 1 and Supplementary Figure 1c), where we did not find any significant differences.

From these results, we concluded that data from the different experiments can be integrated and used for joint analyses of behavioral changes over the lifespan.

Supplementary Table 1. Comparison of GP-UCB parameter estimates across different experiments

**Parameter**	**(Age)**	U	p	r_τ	BF
Generalization λ	(7-9)	390	.930	$-.01$	.29
Generalization λ	(>20)	438	.313	$-.10$	.38
Exploration β	(7-9)	373	.721	$-.04$	.29
Exploration β	(>20)	554	.626	.05	.28
Temperature τ	(7-9)	422	.685	.05	.31
Temperature τ	(>20)	496	.800	$-.03$	.27

Note: We conducted non-parametric two-sample tests to compare the estimates for children between 7-9 from Experiment 1 ($n = 36$) and Experiment 2 ($n = 22$) and the estimates for adult participants older than 20 from Experiment 1 ($n = 24$) and Experiment 3 ($n = 43$).

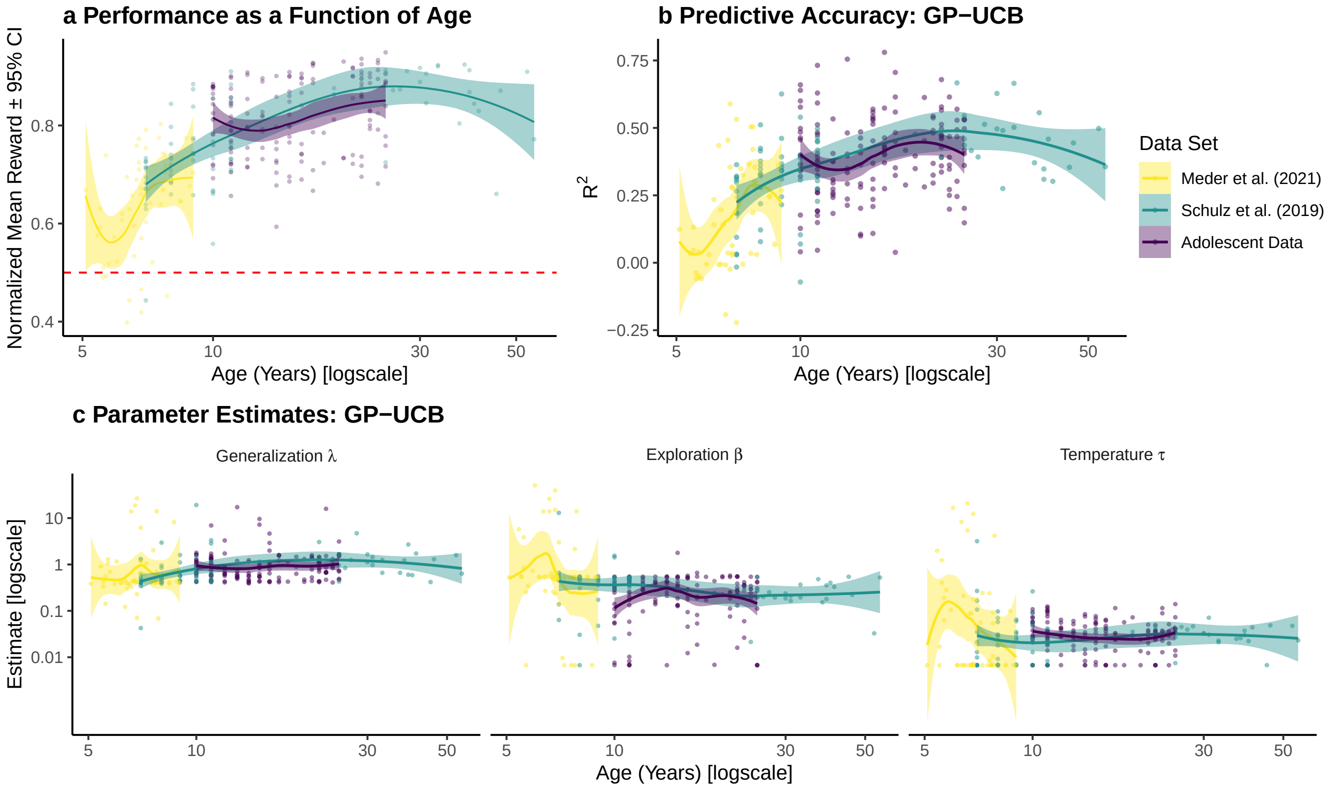

Supplementary Figure 1. Reliability checks. **a** Average reward as a function of age, lines show the smoothed conditional means and 95% confidence interval for data from each experiment. Dots represent the mean reward for each participant and the red dashed line shows the performance of a random choice model. **b**) Predictive accuracy of the GP-UCB model as a function of age and **c**) Parameter estimates of the GP-UCB model as a function of age, separated by experiment. Lines show the smoothed conditional means and 95% confidence interval and dots represent the predictive accuracy per participant.)

2 Statistics

2.1 Comparisons

Both frequentist and Bayesian statistics are reported throughout this paper. Frequentist tests are reported as Student's t-tests (specified as either paired or independent) for parametric comparisons, while the Mann-Whitney U test or Wilcoxon signed-rank test are used for non-parametric comparisons (for independent samples or paired samples, respectively). Each of these tests are accompanied by a Bayes factors (BF) to quantify the relative evidence the data provides in favor of the alternative hypothesis (H_A) over the null (H_0). For parametric comparisons, this is done using the default two-sided Bayesian t-test for either independent or dependent samples, using a Jeffreys-Zellner-Siow prior with its scale set to $\sqrt{2}/2$, as suggested by Ref[1]. For non-parametric comparisons, the Bayesian test is based on performing posterior inference over the test statistics (Kendall's r_τ for the Mann-Whitney-U test and standarized effect size $r = \frac{Z}{\sqrt{N}}$ for the Wilcoxon signed-rank test) and assigning a prior using parametric yoking[2]. This leads to a posterior distribution for for Kendall's r_τ or the standarized effect size r, which yields an interpretable Bayes factor via the Savage-Dickey density ratio test. The null hypothesis posits that the parameters do not differ between the two groups or from the baseline, while the alternative hypothesis posits an effect and assigns an effect size using a Cauchy distribution with the scale parameter set to $1/\sqrt{2}$. All statistical tests are non-directional as defined by a symmetric prior.

2.2 Correlations

For testing linear correlations with Pearson's r, the Bayesian test is based on Jeffreys[3] test for linear correlation and assumes a shifted, scaled beta prior distribution $B(\frac{1}{k}, \frac{1}{k})$ for r, where the scale parameter is set to $k = \frac{1}{3}$, following Ref[4]. For testing rank correlations with Kendall's tau, the Bayesian test is based on parametric yoking to define a prior over the test statistic[5], and performing Bayesian inference to arrive at a posterior distribution for r_τ. The Savage-Dickey density ratio test is used to produce an interpretable Bayes Factor. Note that when performing group comparisons of correlations computed at the individual level, we report the mean correlation and the statistics of a single-sample t-test comparing the distribution of z-transformed correlation coefficients to $\mu = 0$.

3 Supplementary Behavioral results

3.1 Learning over rounds regression

We used a Bayesian hierarchical regression model to analyze if performance changed over multiple rounds of the task. We model participants as random intercepts, with rounds, age groups, and their interaction as fixed effects with random slopes. Supplementary Figure 2 and Supplementary Table 2 provide the model results. They show no effect of round and no reliable interactions between round and age group.

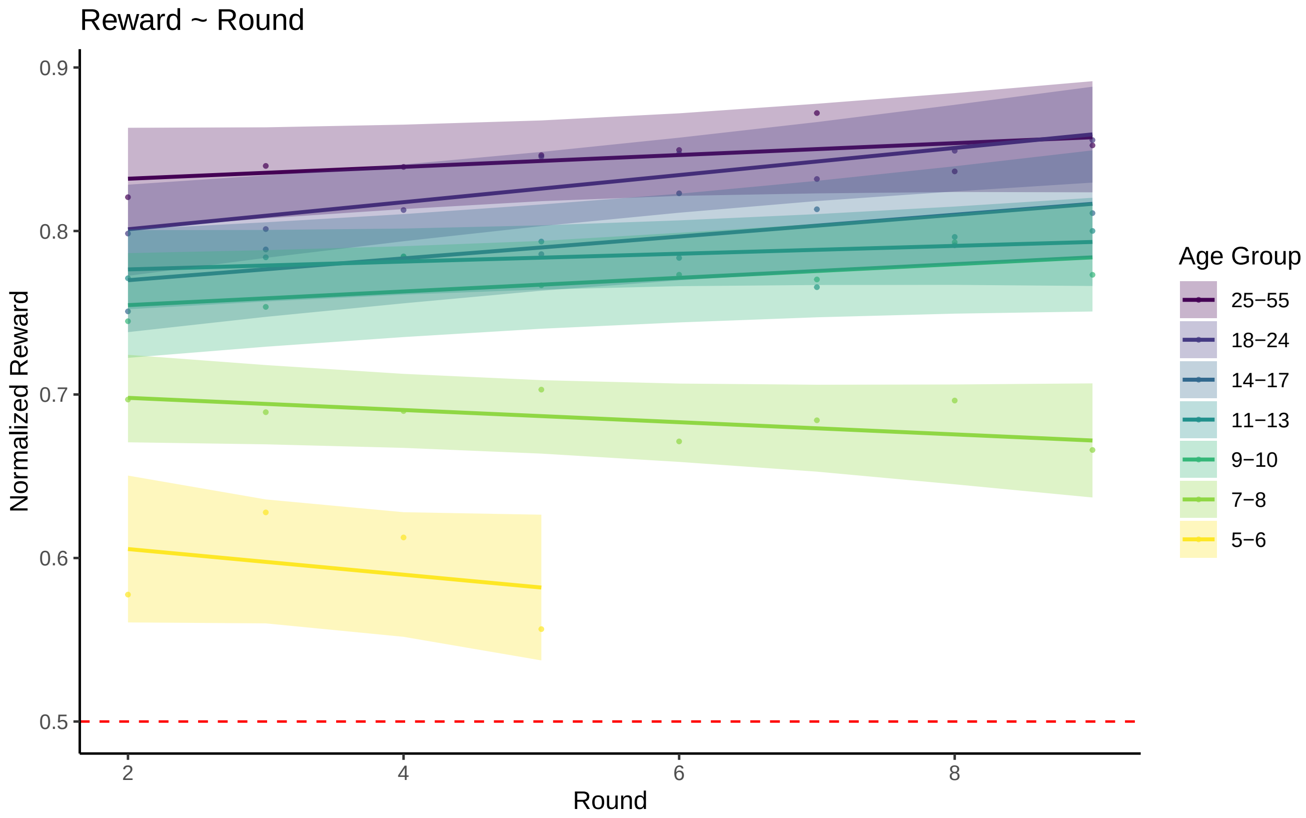

Supplementary Figure 2. Learning over rounds. Reward as a function of rounds. Each line is the fixed effect of a hierarchical Bayesian regression (Supplementary Table 2) with the ribbons indicating 95% CI. Dots show the group mean in each round and the red dashed line indicates a random baseline. We found no effect of round and no reliable interactions between round and age group.

Supplementary Table 2. Bayesian linear multilevel regression: learning over rounds as a function of age group.

	**Estimate**	**95% HDI**
Intercept	0.82	[0.79, 0.86]
Round	0.00	[-0.00, 0.01]
Age group 18-24	-0.04	[-0.09, -0.01]
Age group 14-17	-0.07	[-0.12, -0.01]
Age group 11-13	-0.05	[-0.10, -0.00]
Age group 9-10	-0.08	[-0.13, -0.02]
Age group 7-8	-0.12	[-0.17, -0.07]
Age group 5-6	-0.20	[-0.28, -0.12]
Round× Age group 18-24	0.00	[-0.00, 0.01]
Round × Age group 14-17	0.00	[-0.00, 0.01]
Round × Age group 11-13	-0.00	[-0.01, 0.01]
Round × Age group 9-10	0.00	[-0.01, 0.01]
Round × Age group 7-8	-0.01	[-0.02, 0.00]
Round × Age group 5-6	-0.01	[-0.03, 0.01]
**Random Effects**		
σ^2	0.01	
τ_{00}	0.00	
$\tau_{11\ \text{round}}$	0.00	
$\tau_{11\ \text{age group 18-24}}$	0.00	
$\tau_{11\ \text{age group 14-17}}$	0.00	
$\tau_{11\ \text{age group 11-13}}$	0.00	
$\tau_{11\ \text{age group 9-10}}$	0.00	
$\tau_{11\ \text{age group 7-8}}$	0.00	
$\tau_{11\ \text{age group 5-6}}$	0.01	
ICC	0.28	
N	281	
Observations	2040	
Bayesian R^2	0.48	

Note: Posterior mean and 95% highest density interval (HDI) are reported for all coefficients. Age group 25-55 is the reference level for the categorical variable age group. σ^2 indicates the individual-level variance and τ the variation between individual intercepts and average intercept. ICC is the intraclass correlation coefficient.

3.2 Search distance regression

We used a Bayesian hierarchical linear regression model to analyze the relation between the obtained reward and search distance in the next trial. In this model, participants were treated as random effects. Age group, reward obtained in the previous round, and their interaction were treated as fixed effects with random slopes. Supplementary Table 3 provides the model results. They indicate an effect of age group, older participants showed higher search distances. Additionally, we found an effect of previous reward on search distance, higher rewards lead to sampling of closer tiles. Furthermore, results suggest an interaction between age group and previously obtained reward: older participants adjust their search distance more in line with the previously obtained reward than younger participants do.

Supplementary Table 3. Bayesian linear multilevel regression: search distance as a function of previous reward.

	**Estimate**	**95% HDI**
Intercept	8.35	[7.64, 9.04]
Previous reward	-7.76	[-8.51, -6.99]
Age group 18-24	-0.75	[-1.65, 0.17]
Age group 14-17	-1.25	[-2.18, -0.33]
Age group 11-13	-1.35	[-2.22, -0.44]
Age group 9-10	-2.15	[-3.05, -1.23]
Age group 7-8	-3.91	[-4.82, -3.01]
Age group 5-6	-5.30	[-6.25, -4.36]
Previous reward × Age group 18-24	0.70	[-0.31, 1.67]
Previous reward × Age group 14-17	1.28	[0.27, 2.29]
Previous reward × Age group 11-13	1.47	[0.48, 2.42]
Previous reward × Age group 9-10	2.37	[1.33, 3.39]
Previous reward × Age group 7-8	4.43	[3.45, 5.44]
Previous reward × Age group 5-6	6.32	[5.21, 7.42]
**Random Effects**		
σ^2	3.17	
τ_{00}	3.52	
$\tau_{11 \text{ previous reward}}$	4.24	
$\tau_{11 \text{ age group 18-24}}$	0.04	
$\tau_{11 \text{ age group 14-17}}$	0.16	
$\tau_{11 \text{ age group 11-13}}$	0.10	
$\tau_{11 \text{ age group 9-10}}$	0.33	
$\tau_{11 \text{ age group 7-8}}$	0.05	
$\tau_{11 \text{ age group 5-6}}$	0.40	
ICC	0.11	
N	281	
Observations	51000	
Bayesian R^2	0.40	

Note: Posterior mean and 95% highest density interval (HDI) are reported for all coefficients. Age group 25-55 is the reference level for the categorical variable age group. σ^2 indicates the individual-level variance and τ the variation between individual intercepts and average intercept. ICC is the intraclass correlation coefficient.

4 Supplementary Model Results

Supplementary Table 4. Summary of model results over all participants and separated by age group

**Group**	**Model**	R^2	n	**nLL**	pxp	**Gener.** λ	**Error Var.** $\sqrt{\theta_\varepsilon^2}$	**Explor.** β	**Temp.** τ	**Epsi.** ε
**Overall**										
	GP-UCB	0.34	178	483.94	>.99	0.66		0.38	0.03	
	λ lesion	0.18	23	609.91	<.01		4.3	0.3	0.05	
	β lesion	0.24	47	563.62	<.01	1.66			0.13	
	τ lesion	0.2	33	599.93	<.01	0.32		1.94		0.6
**Age 5-6**										
	GP-UCB	0.11	14	371.15	.60	0.42		0.55	0.07	
	λ lesion	0.02	3	405.8	.07		2.42	12.91	1.03	
	β lesion	0.03	2	401.43	.07	1.43			0.23	
	τ lesion	0.11	11	372.18	.27	0.15		5.23		0.75
**Age 7-8**										
	GP-UCB	0.26	32	478.06	>.99	0.45		0.52	0.02	
	λ lesion	0.05	0	611.19	<.01		2.79	3.94	0.18	
	β lesion	0.1	4	581.7	<.01	1.57			0.18	
	τ lesion	0.17	11	530.1	<.01	0.15		7.17		0.61
**Age 9-10**										
	GP-UCB	0.36	24	525.57	>.99	0.67		0.38	0.03	
	λ lesion	0.17	1	682.01	<.01		3.26	0.29	0.07	
	β lesion	0.25	8	614.91	<.01	1.64			0.14	
	τ lesion	0.21	4	650.85	<.01	0.27		2.18		0.6
**Age 11-13**										
	GP-UCB	0.36	32	533.65	>.99	0.67		0.37	0.03	
	λ lesion	0.17	5	687.85	<.01		4.9	0.28	0.04	
	β lesion	0.26	10	616.47	<.01	1.63			0.12	
	τ lesion	0.19	3	671.15	<.01	0.51		0.54		0.62
**Age 14-17**										
	GP-UCB	0.4	25	498.37	>.99	0.72		0.34	0.03	
	λ lesion	0.25	5	627.3	<.01		4.25	0.26	0.03	
	β lesion	0.29	9	587.46	<.01	1.72			0.1	
	τ lesion	0.23	3	640.55	<.01	0.95		0.3		0.58
**Age 18-24**										
	GP-UCB	0.43	32	470.78	>.99	0.89		0.28	0.03	
	λ lesion	0.28	6	595.40	<.01		5.45	0.28	0.01	
	β lesion	0.34	6	550.87	<.01	1.82			0.07	
	τ lesion	0.24	0	634.72	<.01	1.26		0.22		0.53
**Age 25-55**										
	GP-UCB	0.43	19	471.26	>.99	1.23		0.24	0.03	
	λ lesion	0.29	3	590.8	<.01		3.96	0.24	0.02	
	β lesion	0.36	8	532.53	<.01	2.1			0.07	
	τ lesion	0.22	1	646.14	<.01	1.19		0.21		0.57

Note: We report the average predicted accuracy per model (R^2), the amount of participants best described by the respective model (n), the average out-of-sample negative log likelihood (nLL), the protected exceedance probability (pxp), and median parameter estimates of Generalization λ, Error Variance $\sqrt{\theta_\varepsilon^2}$, Exploration β, Temperature τ, and the epsilon-greedy parameter ε.

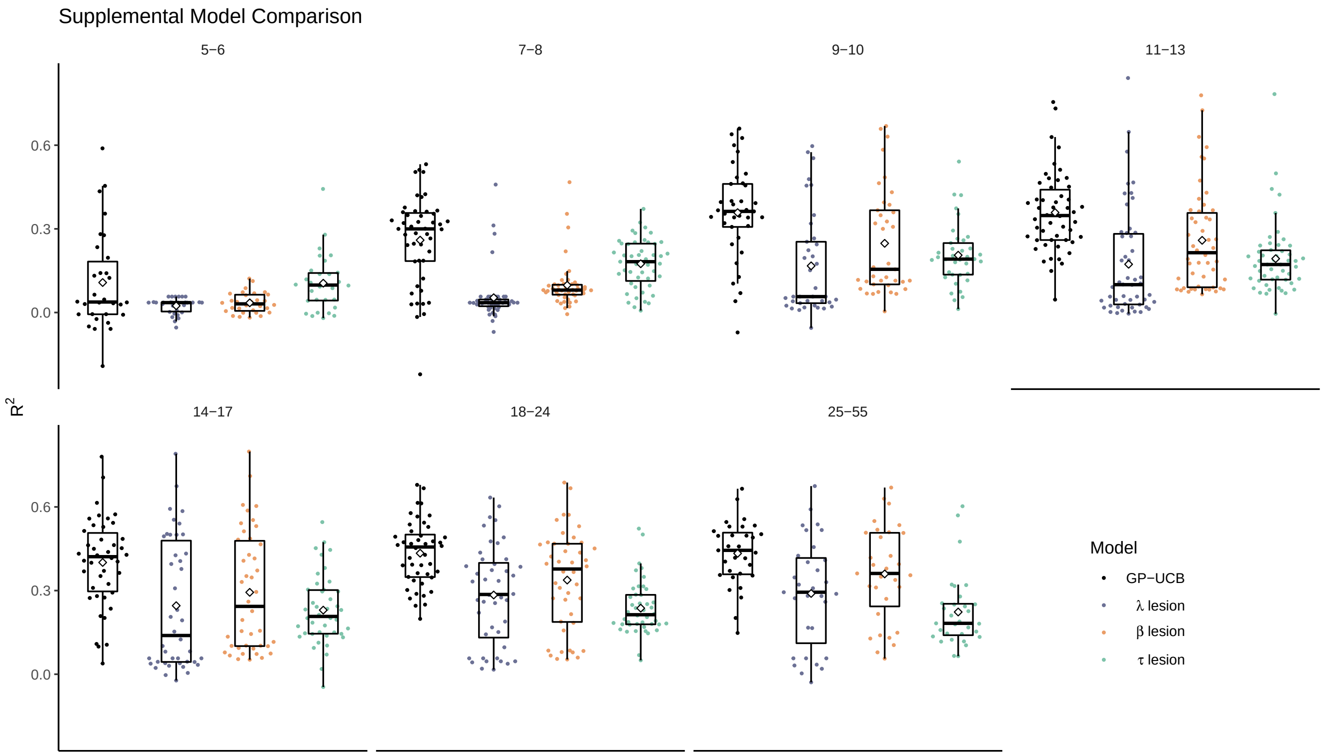

Supplementary Figure 3. Supplemental model comparisons. Tukey's boxplots showing the predictive accuracy for all models and age groups, with box plots indicating the interquartile range (box), the median (horizontal line), mean (diamond) and 1.5-times interquartile range (whiskers). Each dot is the predictive accuracy for one participant. Refer to Figure 2a for sample sizes of age groups.

Model recovery

A prerequisite for interpreting computational models is to ensure each model is uniquely identifiable[6]. To establish that this is the case for models under consideration, we simulated experiments using the subject-level parameter estimates from each model. These simulations were performed using the same set of environments experienced by participants and generated the same data structure as recorded from participants. We then re-estimated each model to the simulated data sets and evaluated how often each model provided the best account of the generated data (based on the summed nLLs over from leave-one-round-out cross-validation). This provides an estimate of the probability with which each model best fits the data given a known simulating model (Supplementary Figure 4 top row; in each plot, the columns sum to 1). Based on these probabilities we also computed the inverse matrix, using Bayes theorem to define the probability that a model in fact generated the data given that observation that it provided the best fit to the data (Supplementary Figure 4 bottom row; in each plot, the rows sum to 1).

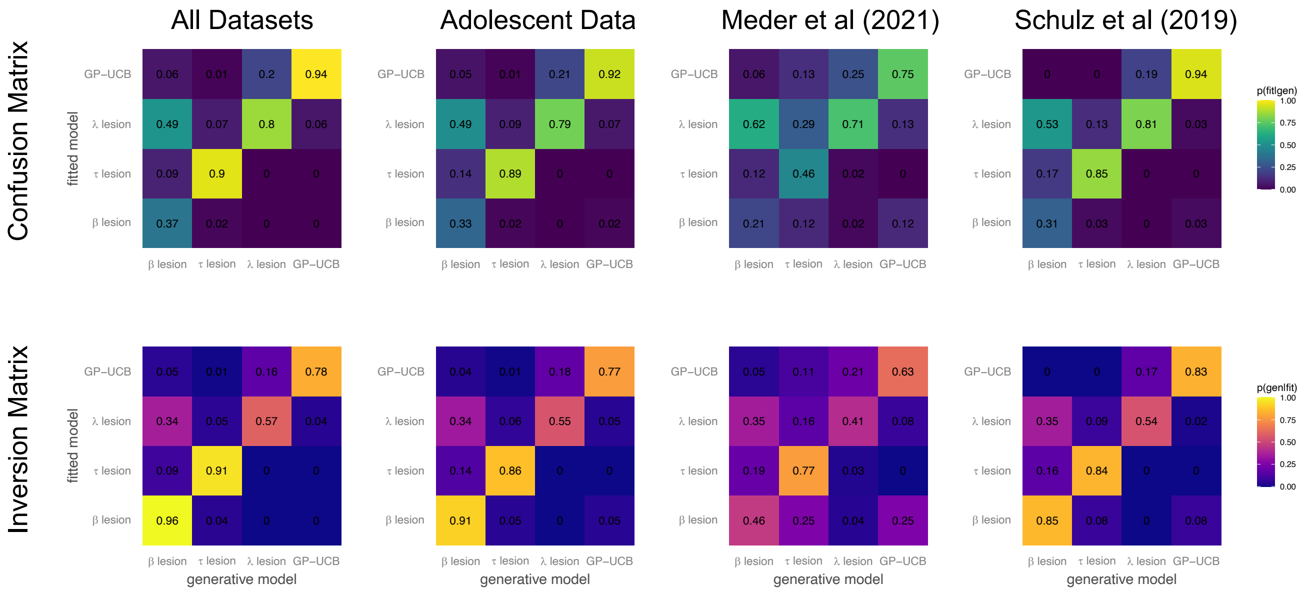

Supplementary Figure 4. Model Recovery. **Top row**: Confusion matrices showing the probability that a model provides the best out-of-sample predictions (y-axes), given an underlying generative model (x-axes), where behavior was generated using participant parameter estimates. Columns sum to 1 (subject to rounding error). **Bottom row**: Inversion matrices showing the probability that a model did in fact generate the data (x-axes), given that it was found to provide the best out-of-sample predictions (y-axes). Rows sum to 1 (subject to rounding error). The first column refers to all experiments combined, subsequent columns show individual experiments

4.1 Parameter recovery

We simulated choices using the winning GP-UCB in order to establish whether the models' parameter estimates are reliable. We evaluated the recoverability of the GP-UCB parameters based on two procedures.

First, following ref[6], we produced a synthetic data-set by simulating choices the base of all empirical subject-level parameter estimates obtained from fitting model to the adolescent data set (Figure 5a; for recovery analyses of the other data-sets see[7]). In all cases, recovered parameters were highly correlated with the parameters used to generate the data (all $r_\tau > .91$, $p < .001$, $BF > 100$).

Second, we performed a more rigorous parameter recovery by iteratively varying each parameter of the GP-UCB model over 20 linearly spaced values within a credible interval of parameter estimates in all experiments (using Tukeys' fence). This creates a set of counter-factual parameters to test whether we can recover parameters we did not originally estimate from the data. Again, we find a high degree of correlation between recovered and generating parameter value (all $r_\tau > .86$, $p < .001$, $BF > 100$).

While the first parameter recovery provides evidence that empirically observed parameter values are recoverable, the second analysis provides additional evidence that even counterfactual parameters (within a credible interval across all datasets) are also recoverable.

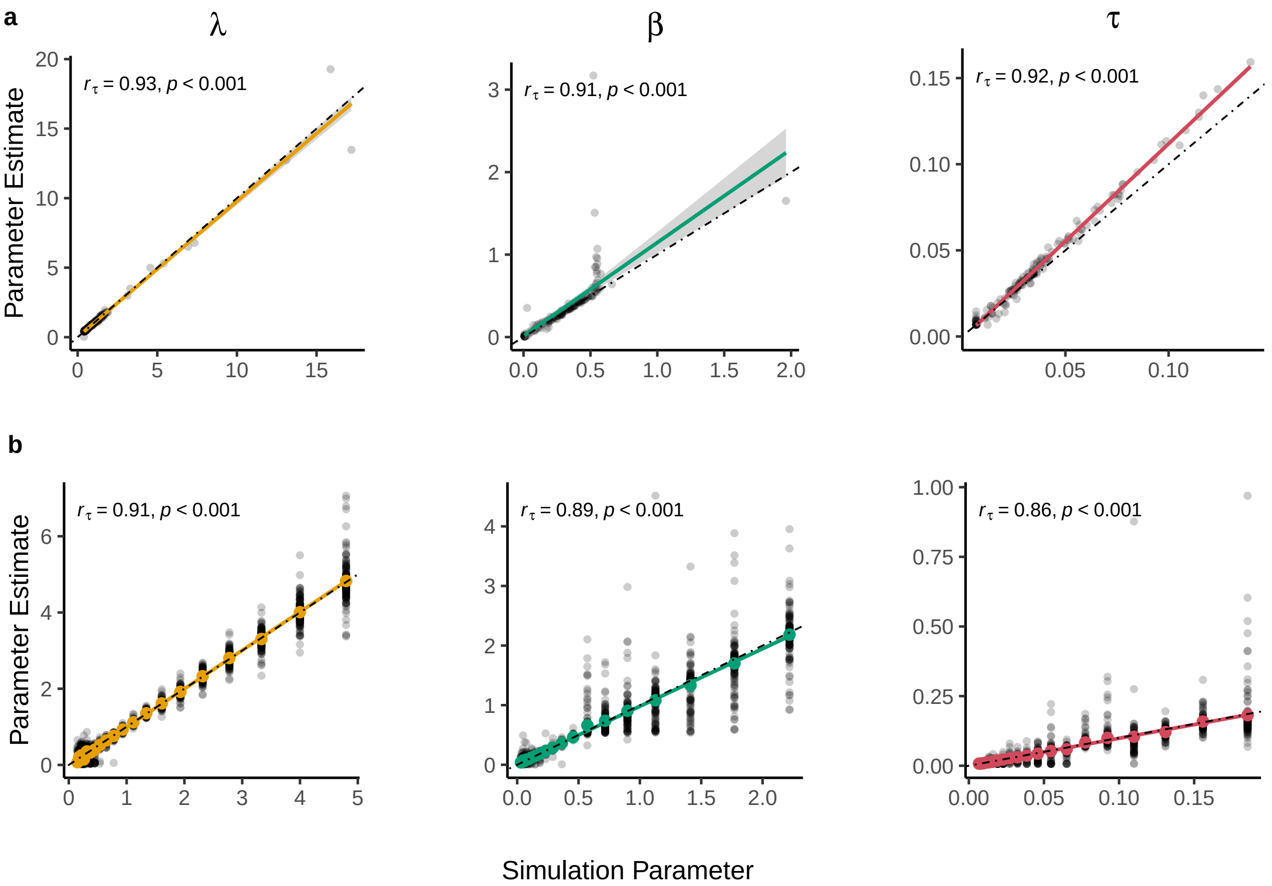

Supplementary Figure 5. Parameter recovery (GP-UCB). **a**) Parameter recovery using participant parameter estimates to generate data. The black dots denote individual parameter estimates. The colored lines show linear fit, while the dotted line denotes a diagonal indicative of perfect recovery. **b**) Augmented parameter recovery, using participant parameters but with systematic variation across a log-space grid of plausible counterfactual parameter values. This provides additional robustness by simulating data across a wider range of possible parameters. Black dots denote each simulated and generating parameter, while the colored dots the mean 95%CI. The colored lines show linear fit, while the dotted line denotes a perfect recovery. For all subplots, the insetted statistics refer to the rank correlation between generative and fitted parameters, Kendalls τ.

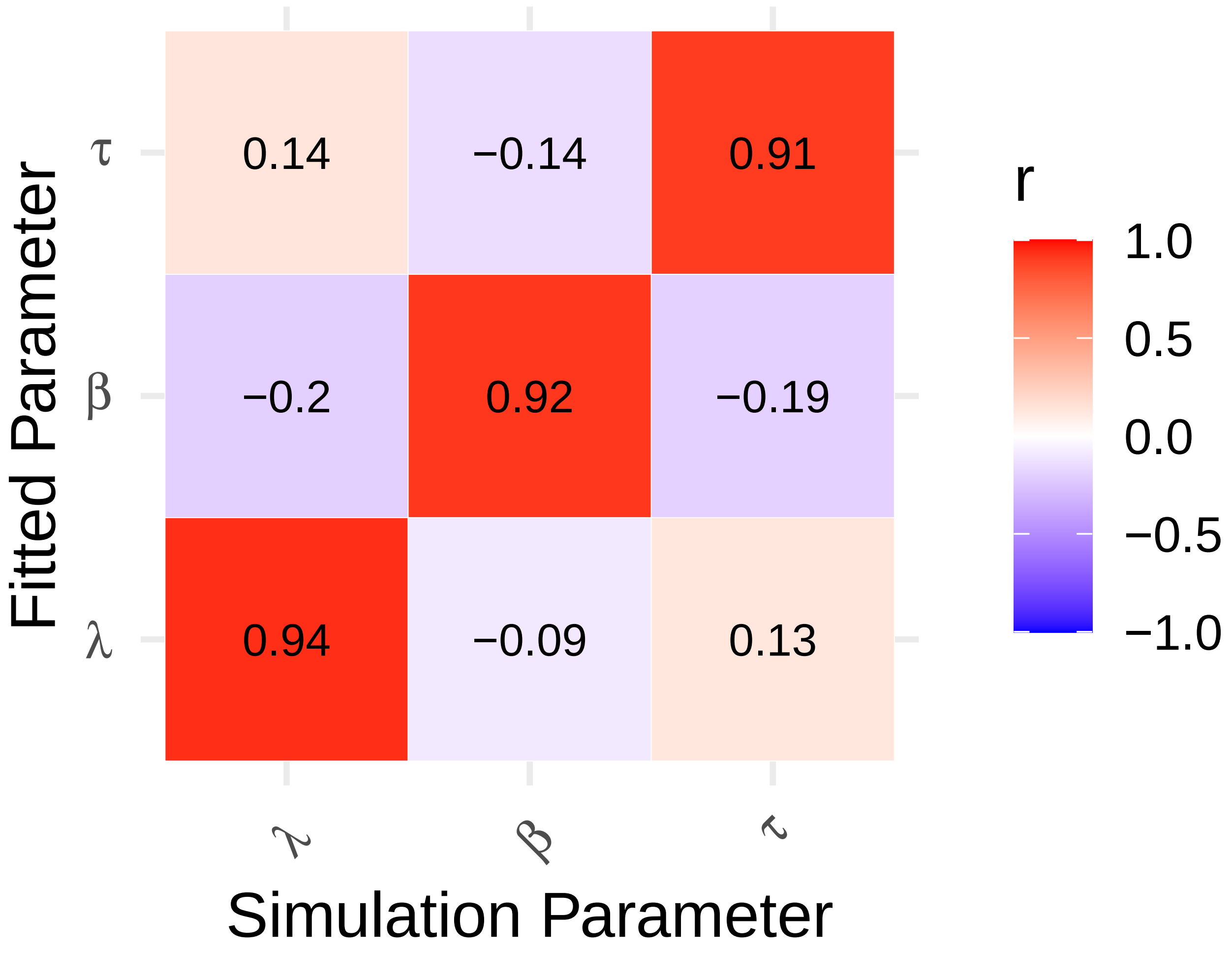

Supplementary Figure 6. Parameter recovery (GP-UCB). **a**) Estimated parameters correlate strongly with their counterparts used for simulation (diagonal) while correlations with other parameters used for simulation (off-diagonal) are weak.

4.2 Comparison of Parameter Regression Models

For all regression models quantifying the relationship between the GP-UCB parameters and age we computed approximate leave-one-out cross validation using pareto-importance sampling as described in Ref[8]. We then used the resulting expected log pointwise predictive densities as model selection criterion. We included models that predicted log-transformed and untransformed parameters. Due to the bounded range of parameter values (>0), log-transformation resulted in more accurate regression models.

Supplementary Table 5. Comparison of Parameter Regression Models

Model Class	Transformation	elpd_diff	se_diff	elpd_loo	se_elpd_loo	p_loo	se_p_loo	looic	se_looic
Changepoint	log	0.000000	0.000000	-1300.948	50.48478	33.61886	6.419699	2601.897	100.96956
4th degree polynomial	log	-7.645817	3.898103	-1308.594	49.93090	32.89042	6.255166	2617.188	99.86179
3rd degree polynomial	log	-8.591376	5.105124	-1309.540	50.65230	31.13398	6.143772	2619.079	101.30460
2nd degree polynomial	log	-10.876295	6.552031	-1311.825	51.54652	28.73522	5.991910	2623.649	103.09304
Linear	log	-13.945475	7.652849	-1314.894	52.22692	25.43520	5.634014	2629.788	104.45383
Changepoint	none	-614.963523	147.352916	-1915.912	178.84787	127.23947	63.851037	3831.824	357.69573
4th degree polynomial	none	-616.156643	145.789407	-1917.105	177.06655	120.84630	60.607442	3834.210	354.13309
2nd degree polynomial	none	-616.415606	146.075088	-1917.364	177.57634	120.07705	60.607337	3834.728	355.15269
3rd degree polynomial	none	-618.210471	146.807664	-1919.159	178.14811	127.37190	65.709042	3838.318	356.29623
Linear	none	-620.134252	148.303877	-1921.083	179.86334	119.26099	60.089972	3842.165	359.72667

Note: Comparison of regression models predicting log-transformed parameter estimates as a function of age. Models are described in descending order of fit (best models first). elpd_diff describes the difference in expected log point-wise predictive density, relative to the model with the best predictive accuracy, while se_diff, denotes the standard error of the difference. elpd_loo describes the Bayesian LOO estimate of the expected log pointwise predictive density (Eq 4 in Ref[8]), which is the sum of $n = 281$ pointwise predictive densities, with se_elpd_loo denoting the standard error. p_loo, is the difference between the elpd-loo and the non-cross-validated predictive density, with se_p_loo denoting the standard error. Lastly, looic is the loo information criterion $-2*\text{elpd}$ and can be interpreted similarly to other information criteria such as BIC or AIC, with se_looic denoting its standard error.

4.3 Lesioned Parameter Regression Models

Following the same procedure as above, we also computed approximate leave-one-out-cross-validation of regression models that constrain the changepoints to some parameters, while other parameters were treated as an intercept-only model. To do so, we used the multivariate syntax implemented in brms[9], allowing us to implement any combination of changepoint and intercept-only models for all parameters. The first row denotes the parameter that was fitted using an intercept-only model. The other parameters were fitted as a changepoint model, according to Equation 14.

Supplementary Table 6. Comparison of Lesioned Parameter Regression Models

Model Class	elpd_diff	se_diff	elpd_loo	se_elpd_loo	p_loo	se_p_loo	looic	se_looic
No intercept only	0.000000	0.000000	-1300.558	50.35418	33.29739	6.355287	2601.117	100.70836
λ intercept only	-3.862616	4.691819	-1304.421	49.35334	29.56012	5.826601	2608.842	98.70668
τ intercept only	-7.336907	6.778480	-1307.895	53.07852	29.10912	5.851021	2615.790	106.15704
β intercept only	-9.909657	6.369223	-1310.468	52.48454	29.38624	6.112272	2620.936	104.96908
λ_β intercept only	-10.228406	6.597255	-1310.787	51.65019	26.63042	5.769139	2621.573	103.30039
λ_τ intercept only	-16.958107	8.738406	-1317.516	52.08509	26.51306	5.760537	2635.033	104.17018
β_τ intercept only	-17.402415	8.865649	-1317.961	53.73732	26.66973	6.113856	2635.921	107.47463

Note: Comparison of regression models predicting log-transformed parameter estimates as a function of age. Models are described in descending order of fit (best models first). elpd_diff describes the difference in expected log point-wise predictive density, relative to the model with the best predictive accuracy, while se_diff, denotes the standard error of the difference. elpd_loo describes the Bayesian LOO estimate of the expected log pointwise predictive density (Eq 4 in Ref[8]), which is the sum of $n = 281$ pointwise predictive densities, with se_elpd_loo denoting the standard error. p_loo, is the difference between the elpd-loo and the non-cross-validated predictive density, with se_p_loo denoting the standard error. Lastly, looic is the loo information criterion $-2*\text{elpd}$ and can be interpreted similarly to other information criteria such as BIC or AIC, with se_looic denoting its standard error.

4.4 Parameter Regression Models

Supplementary Table 7. Parameters of the changepoint regression models on participant data

Model	Parameter	Estimate	Est.Error	l-95% CI	u-95% CI	Rhat	Bulk_ESS	Tail_ESS
λ (Generalization)								
	Intercept	-0.11	0.12	-0.36	0.12	1	2938	3158
	b1	0.08	0.07	0.02	0.26	1	2288	930
	b2	0.01	0.01	-0.01	0.02	1	4060	5689
	ω	12.70	0.64	7.60	19.80	1	2141	1158
β (Directed Exploration)								
	Intercept	-1.48	0.15	-1.78	-1.19	1	4192	5086
	b1	-0.39	0.17	-0.79	-0.13	1	4252	3638
	b2	0.00	0.01	-0.03	0.02	1	4961	5561
	ω	9.10	0.23	7.44	11.40	1	4087	4767
τ (Random exploration)								
	Intercept	-3.67	0.14	-3.92	-3.42	1	5423	5993
	b1	-0.59	0.20	-1.05	-0.25	1	5266	4086
	b2	0.01	0.01	-0.01	0.03	1	5790	5385
	ω	7.74	0.08	6.88	8.77	1	4513	3495

Note: The models were fit using Hamiltonian Monte-Carlo sampling with 4 Markov chains, each drawing 4000 samples, 2000 of which were discarded as warm-up. The first column (Estimate) refers to the maximum a posteriori estimate of the respective parameters. The second column (Est.Error) denotes the standard deviation of the posterior. The third and fourth column denote the lower and upper credible interval of the posterior. The fifth column denotes the Gelman Rubin Statistic (Rhat) indicating chain convergence. The sixth and seventh columns shows the number of effective samples from the bulk and the tail of the posterior.

Supplementary Table 8. Parameters of the changepoint regression models on SHC-Fast trajectory

Model	Parameter	Estimate	Est.Error	l-95% CI	u-95% CI	Rhat	Bulk_ESS	Tail_ESS
λ (Generalization)								
	Intercept	0.25	0.02	0.22	0.28	1	1708	1898
	b1	4.84	0.19	4.46	5.23	1	2286	1870
	b2	0.10	0.07	-0.03	0.23	1	1815	1905
	ω	310.35	762.52	294.09	326.21	1	1575	1989
β (Directed Exploration)								
	Intercept	-1.15	0.01	-1.17	-1.13	1	2178	2245
	b1	-2.78	0.35	-3.49	-2.10	1	2019	2106
	b2	0.00	0.04	-0.08	0.08	1	2539	2234
	ω	178.14	787.11	145.89	207.77	1	1250	1623
τ (Random exploration)								
	Intercept	-3.97	0.02	-4.01	-3.93	1	1452	1469
	b1	-3.14	0.31	-3.74	-2.54	1	2029	2385
	b2	0.30	0.07	-0.43	-0.15	1	2066	1957
	ω	255.91	777.67	229.35	293.56	1	1284	1354

Note: The models were fit using Hamiltonian Monte-Carlo sampling with 4 Markov chains, each drawing 4000 samples, 2000 of which were discarded as warm-up. The first column (Estimate) refers to the maximum a posteriori estimate of the respective parameters. The second column (Est.Error) denotes the standard deviation of the posterior. The third and fourth column denote the lower and upper credible interval of the posterior. The fifth column denotes the Gelman Rubin Statistic (Rhat) indicating chain convergence. The sixth and seventh columns shows the number of effective samples from the bulk and the tail of the posterior.

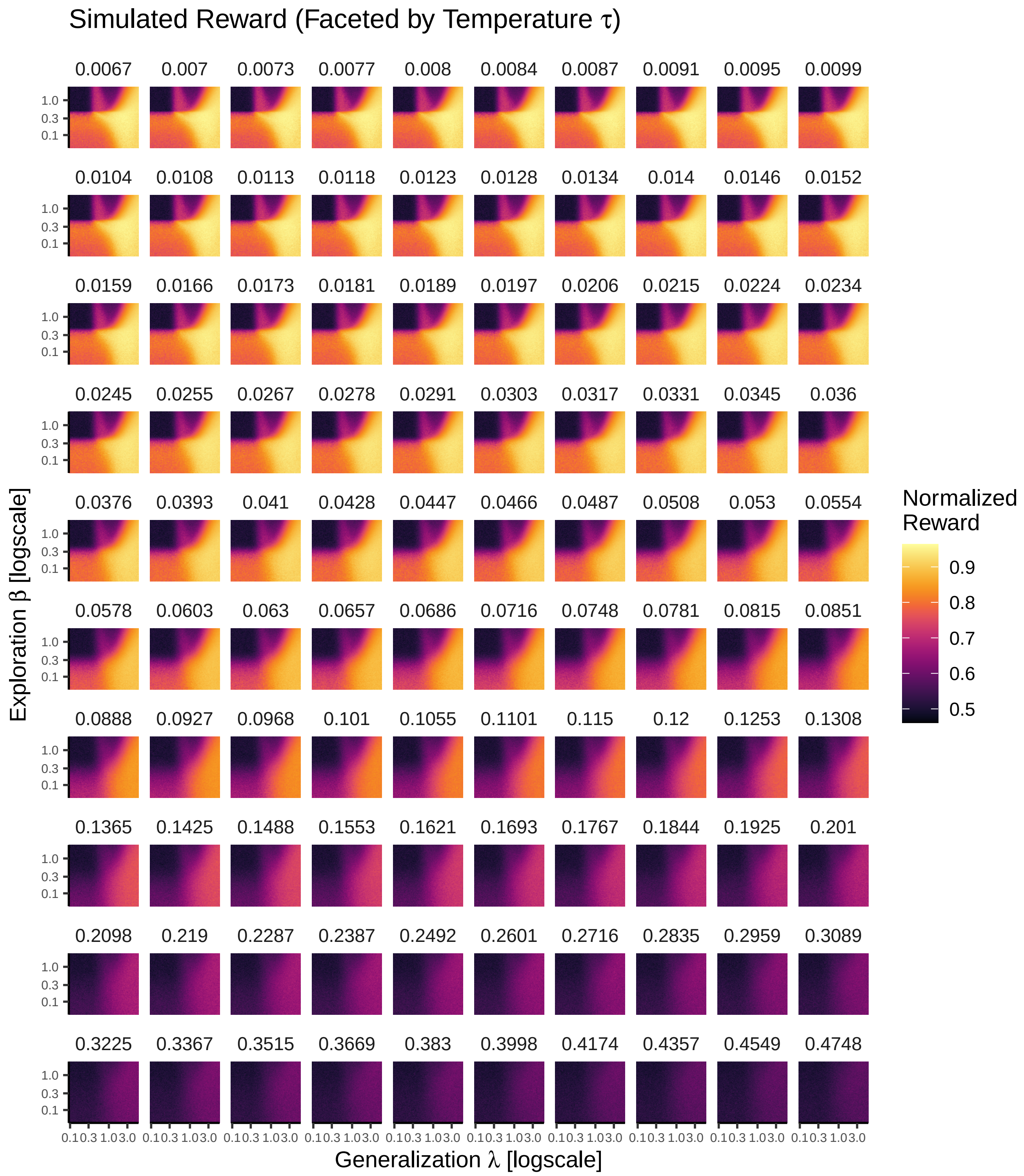

Supplementary Figure 7. Fitness landscape. The 3-dimensional fitness landscape is computed over 1 million parameter combinations across λ (x-axis), β (y-axis), and τ (facets). For each parameter, we defined 100 equally log-spaced values over a credible range of participant parameter estimates (using Tukey's fence). We then simulated 100 rounds of the task using each combination of parameters (sampling with replacement from the same set of 40 environments given to participants) in order to compute an average reward, which we then normalized to a max of 1.

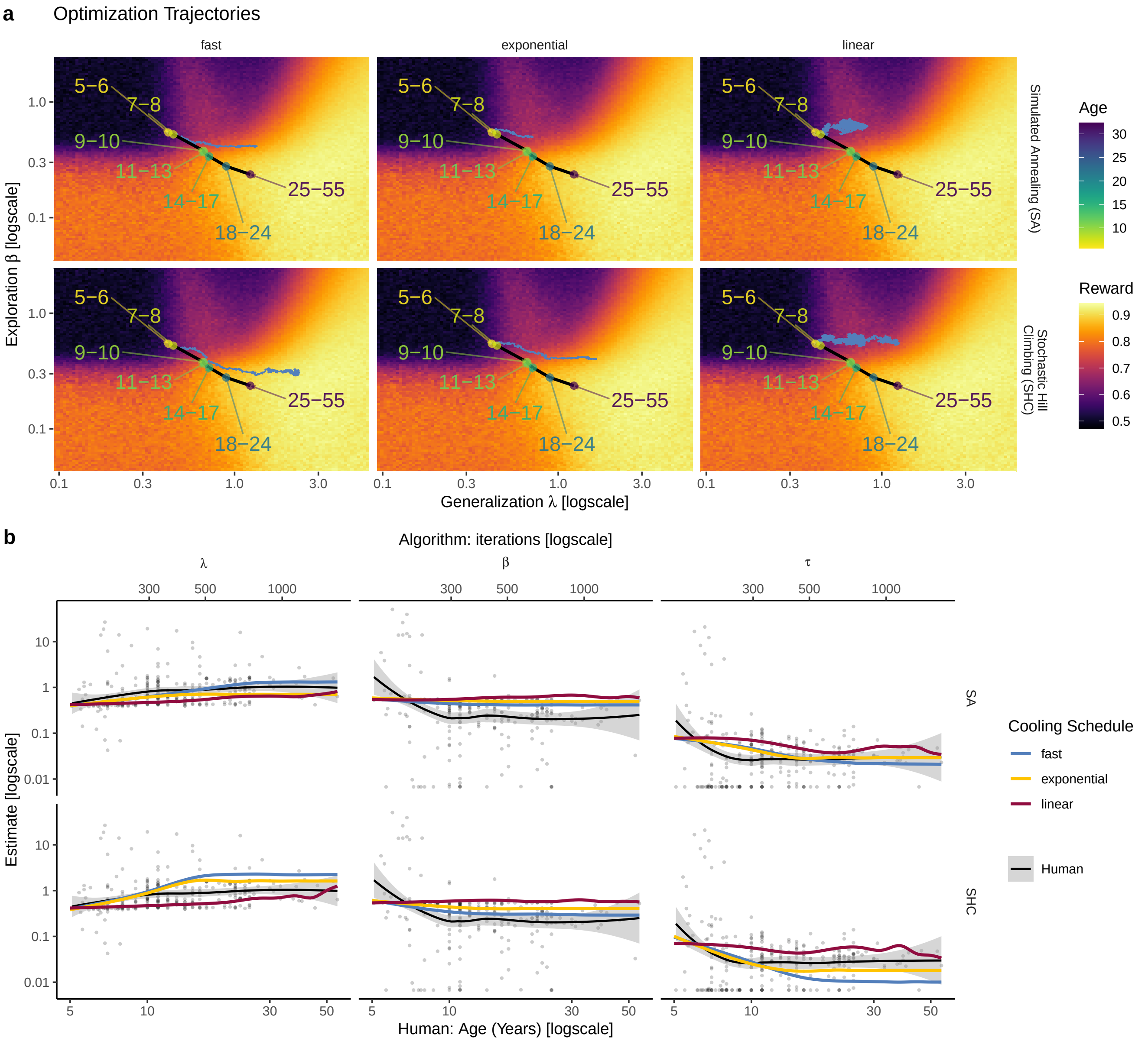

Supplementary Figure 8. Supplementary results of the optimization algorithms. **a**) Trajectories of λ and β for each combination of optimization algorithm (rows) and cooling function (columns). For the background, we used the τ value with the smallest difference to the mean simulated τ value across all trajectories and iterations ($\bar{\tau} = .03$). Human estimates (labeled dots) are also provided for comparison. **b**) Comparison of the human developmental trajectory (black line indicates smoothed means ± 95% CI and dots show each individual parameter) to each optimization algorithm (colored lines). Human data is plotted along age in years (bottom axis), while the algorithm results are plotted in terms of iteration number (top axis), both in log scale.

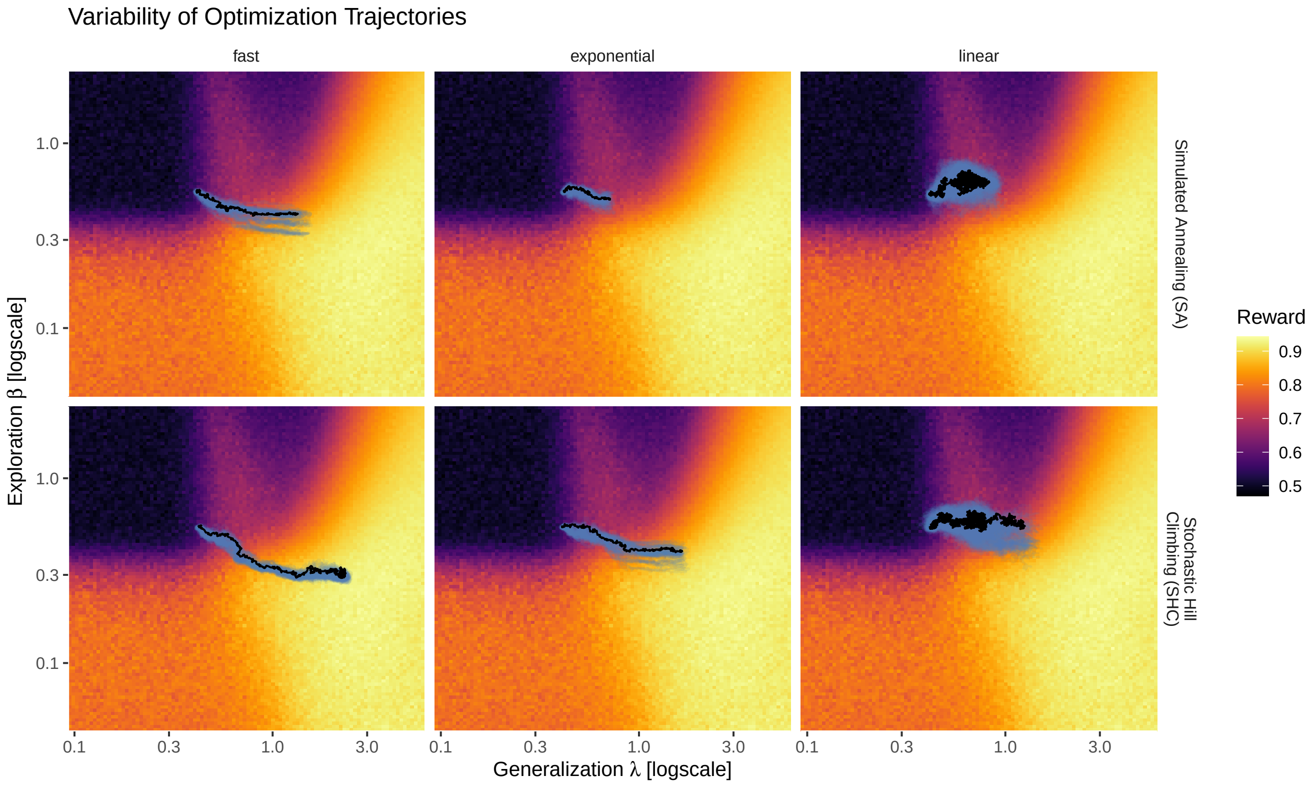

Supplementary Figure 9. Variability of trajectories of generalization λ and uncertainty-directed exploration β. Plots show the bootstrapped variability of trajectories (blue) as well as the median trajectories (black) for each combination of optimization algorithm (rows) and cooling function (columns). Each combination of algorithm and cooling schedule was initialized with all individual cross-validated parameter estimates of the youngest age group once (30 participants $\times$ 4 rounds), which provides us with four trajectories per participant and algorithm. To visualize the variability of algorithm trajectories, we iteratively dropped one of the trajectories for each participant and then use the same aggregation method (mean for each participant and then the median trajectory across participants; 100 iterations). We then plot each of these leave-one-out aggregated trajectories to visualize the variability of the algorithm trajectories.

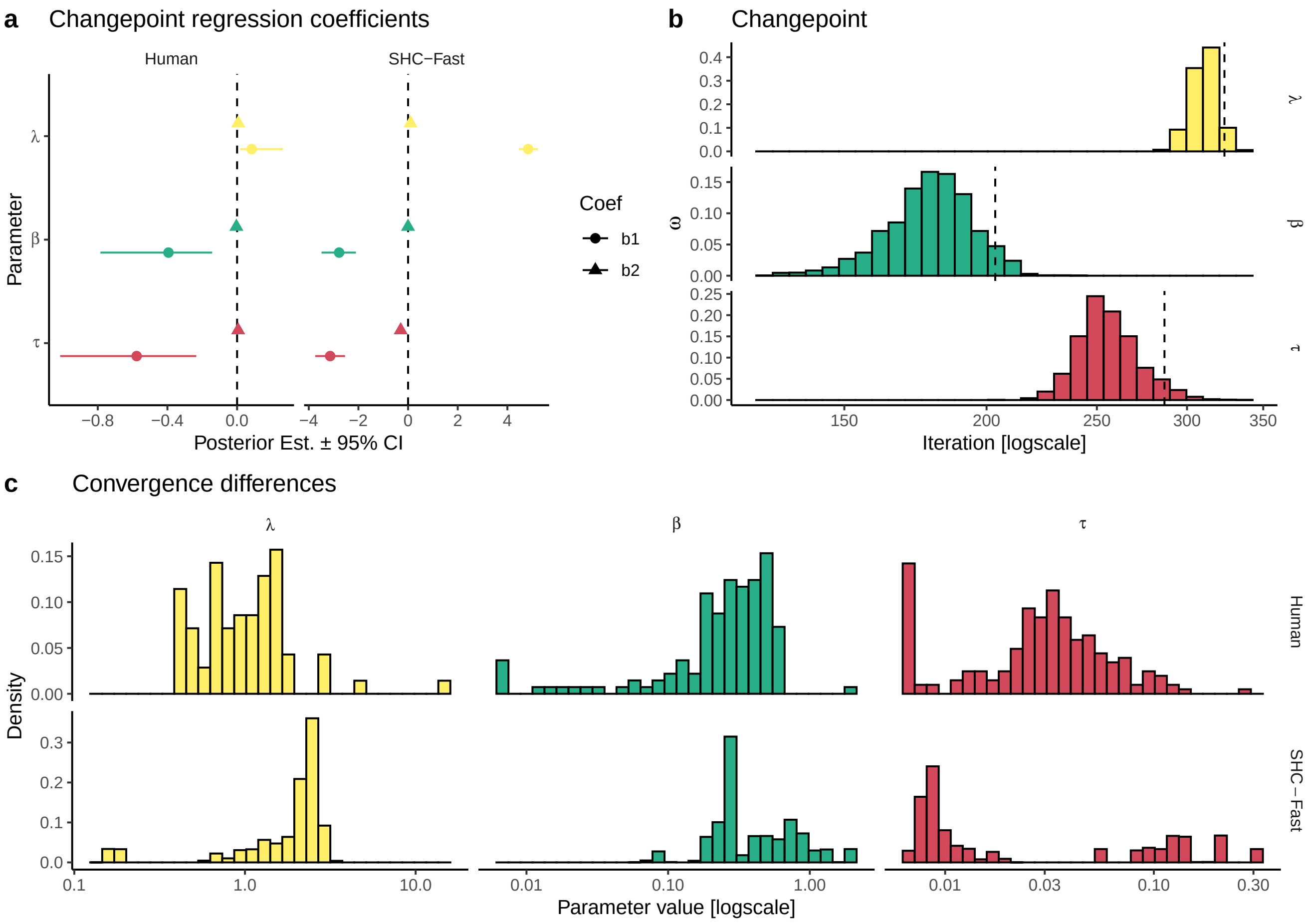

Supplementary Figure 10. Similarity between human and optimization algorithm. To compute how human and algorithm trajectories differ, we first generated 100 bootstrapped datasets of the SHC-fast cooling trajectories, matched in size to our participant data ($n = 281$). This allows us to run a comparable version of the changepoint regression, comparing humans to the algorithm. Specifically, we first created a unified time variable for human and algorithm trajectories by normalizing participant age and algorithm iterations to the same range of $[0,1]$, respectively. Then for each (unnormalized) age bin in years [5,6, . . ., 55], we randomly sampled (without replacement) the same number of parameters (λ, β, τ) from the algorithm trajectory within the same unified time range. This created a matched dataset of the algorithmic trajectory with the same number of $n = 281$ observations along the same unified time axis. This was repeated 100 times to minimize sampling bias. We than re-ran the change-point regression on this bootstrapped data, using the same formulation as in Eq. 14-15. This allows us to compare b_1 and b_2 between humans and the algorithm trajectories, which yielded qualitatively similar trends (see Supplementary Table 8). **a**) The posterior regression weights of the changepoint regression estimated on both human (left) and algorithm trajectories (SHC-Fast cooling; right). The dots indicate the mean and point-ranges denote the 95% credible interval (CI), while the vertical dashed line indicates 0 (i.e., no change). **b**) Posterior estimates of the changepoint ω from the algorithm trajectories. The vertical dashed line indicates the upper 95% CI. **c**) Parameter distributions after the changepoint (thresholded at the upper 95% CI of the respective ω estimates). The upper panel shows human parameters and the lower panel shows parameters sampled by the SHC algorithm with fast cooling. Human λ and β parameters converged at lower values than the algorithm (λ: $U = 2740$, $p < .001$, $r_\tau = -.38$, $BF > 100$; β: $U = 10114$, $p < .001$, $r_\tau = -.19$, $BF > 100$), while τ estimates were not reliably higher or lower ($U = 22377$, $p = .188$, $r_\tau = .05$, $BF = .12$).

References

1. Rouder, J. N., Speckman, P. L., Sun, D., Morey, R. D. & Iverson, G. Bayesian t tests for accepting and rejecting the null hypothesis. *Psychon. Bull. & Rev.* **16**, 225–237 (2009).
2. van Doorn, J., Ly, A., Marsman, M. & Wagenmakers, E.-J. Bayesian latent-normal inference for the rank sum test, the signed rank test, and Spearman's ρ. *arXiv preprint arXiv:1712.06941* (2017).
3. Jeffreys, H. *The Theory of Probability* (Oxford, UK: Oxford University Press, 1961).
4. Ly, A., Verhagen, J. & Wagenmakers, E.-J. Harold jeffreys's default bayes factor hypothesis tests: Explanation, extension, and application in psychology. *J. Math. Psychol.* **72**, 19–32 (2016).
5. van Doorn, J., Ly, A., Marsman, M. & Wagenmakers, E.-J. Bayesian inference for kendall's rank correlation coefficient. *The Am. Stat.* **72**, 303–308 (2018).
6. Wilson, R. C. & Collins, A. G. Ten simple rules for the computational modeling of behavioral data. *Elife* **8**, e49547 (2019).
7. Schulz, E., Wu, C. M., Ruggeri, A. & Meder, B. Searching for rewards like a child means less generalization and more directed exploration. *Psychol. Sci.* **30**, 1561–1572 (2019). DOI 10.1177/0956797619863663.
8. Vehtari, A., Gelman, A. & Gabry, J. Practical bayesian model evaluation using leave-one-out cross-validation and WAIC. *arXiv [stat.CO]* (2015).
9. Bürkner, P.-C. Advanced Bayesian multilevel modeling with the R package brms. *The R J.* **10**, 395–411 (2018). DOI 10.32614/RJ-2018-017.
