# Supplementary material for: Developmental changes in exploration resemble stochastic optimization
Source: Nat Hum Behav. 2023 Aug 17;7(11):1955–67. doi: 10.1038/s41562-023-01662-1 (PMC10663152; doi:10.1038/s41562-023-01662-1)
Supplement: Supplementary file 2 — Reporting Summary [file 41562_2023_1662_MOESM2_ESM.pdf]

nature portfolio

Corresponding author(s): Dr. Charley Wu

Last updated by author(s): Jun 8, 2023

Reporting Summary

Nature Portfolio wishes to improve the reproducibility of the work that we publish. This form provides structure for consistency and transparency in reporting. For further information on Nature Portfolio policies, see our Editorial Policies and the Editorial Policy Checklist.

Statistics

For all statistical analyses, confirm that the following items are present in the figure legend, table legend, main text, or Methods section.

n/a	Confirmed	
☐	☒	The exact sample size (*n*) for each experimental group/condition, given as a discrete number and unit of measurement
☐	☒	A statement on whether measurements were taken from distinct samples or whether the same sample was measured repeatedly
☐	☒	The statistical test(s) used AND whether they are one- or two-sided<br>*Only common tests should be described solely by name; describe more complex techniques in the Methods section.*
☐	☒	A description of all covariates tested
☐	☒	A description of any assumptions or corrections, such as tests of normality and adjustment for multiple comparisons
☐	☒	A full description of the statistical parameters including central tendency (e.g. means) or other basic estimates (e.g. regression coefficient) AND variation (e.g. standard deviation) or associated estimates of uncertainty (e.g. confidence intervals)
☐	☒	For null hypothesis testing, the test statistic (e.g. *F*, *t*, *r*) with confidence intervals, effect sizes, degrees of freedom and *P* value noted<br>*Give P values as exact values whenever suitable.*
☐	☒	For Bayesian analysis, information on the choice of priors and Markov chain Monte Carlo settings
☐	☒	For hierarchical and complex designs, identification of the appropriate level for tests and full reporting of outcomes
☐	☒	Estimates of effect sizes (e.g. Cohen's *d*, Pearson's *r*), indicating how they were calculated

Our web collection on statistics for biologists contains articles on many of the points above.

Software and code

Policy information about availability of computer code

Data collection	We used online experiments, where experiment code is freely available at https://github.com/charleywu/gridsearch and https://github.com/ericschulz/kwg. Cordova version xy was used to implement the experiment.
Data analysis	All code used to analyze the data is freely available at https://github.com/AnnaGiron/developmental_trajectory. We used R version 4.0.3 and Python 3.8 for the analyses.

For manuscripts utilizing custom algorithms or software that are central to the research but not yet described in published literature, software must be made available to editors and reviewers. We strongly encourage code deposition in a community repository (e.g. GitHub). See the Nature Portfolio guidelines for submitting code & software for further information.

Data

Policy information about availability of data

All manuscripts must include a data availability statement. This statement should provide the following information, where applicable:

- Accession codes, unique identifiers, or web links for publicly available datasets
- A description of any restrictions on data availability
- For clinical datasets or third party data, please ensure that the statement adheres to our policy

Experiment data is freely available at https://github.com/AnnaGiron/developmental_trajectory.

Research involving human participants, their data, or biological material

Policy information about studies with human participants or human data. See also policy information about sex, gender (identity/presentation), and sexual orientation and race, ethnicity and racism.

Reporting on sex and gender	We did not perform sex- or gender-based analysis because we did not expect any sex- or gender-based differences.
Reporting on race, ethnicity, or other socially relevant groupings	We did not collect any data on race, ethnicity or other socially relevant groupings.
Population characteristics	See below.
Recruitment	Participants from Meder et al. (2021) and Schulz et al. (2019) were recruited in museums in Berlin. The new adolescent participants performed the task in the Max Planck Institute for Human Development in Berlin.
Ethics oversight	All studies were approved by the ethics board of the Max Planck Institute for Human Development in Berlin.

Note that full information on the approval of the study protocol must also be provided in the manuscript.

Field-specific reporting

Please select the one below that is the best fit for your research. If you are not sure, read the appropriate sections before making your selection.

☐ Life sciences ☒ Behavioural & social sciences ☐ Ecological, evolutionary & environmental sciences

For a reference copy of the document with all sections, see nature.com/documents/nr-reporting-summary-flat.pdf

Behavioural & social sciences study design

All studies must disclose on these points even when the disclosure is negative.

Study description	The data presented here are a combination of three different datasets (Schulz et al., 2019, Meder et al., 2021 and an unpublished dataset). In all experiments, we collected quantitative data based on individual's choice in each trial. Participants were first given instructions for the task along with several examples of fully revealed environments. Then they were asked to complete a set of comprehension questions before starting the experiment.
Research sample	The data were collected in three different experiments, two of which have been published before (Meder el at., 2021 and Schulz et al., 2019) and one unpublished dataset targeting adolescent participants. Participants from the unpublished dataset completed the task at the Max Planck Institute for Human Development in Berlin along with a battery of 10 other decision-making tasks on a desktop computer. These participants were given a fixed payment of 10€ per hour. The sample size consists of 281 participants between the ages of 5 and 55 (mean age=14.46, sd=8.61, 126 female).
Sampling strategy	The sample size was chosen to be comparable to the previously published datasets (Schulz et al., 2019, Meder et al., 2021) and appropriately scaled for the larger age range. Since the focus of our analysis was on computational modeling, rather than purely behavioral analysis, our sample size determination was not focused on achieving the necessary power to observe a specific effect size.
Data collection	Data from Schulz et al. (2019) and Meder et al (2021) were collected in museums in Berlin and participants performed the task on a tablet. Participants from the unpublished dataset were recruited via phone interview from the internal database of the Max Planck Institute for Human Development. The experimenter explained the task and then left the room while participants performed the experiment along with a battery of 10 other decision-making experiments on a desktop computer. For all datasets, the experimenter was blinded regarding to the study hypothesis.
Timing	The new data was collected between October 2018 and June 2019.
Data exclusions	In the original Meder et al., dataset, 14 participants were excluded due to failing the instruction check (n=9), did not complete the task (n=1), were not native speakers (n=2), or because their parents intervened during the experiment (n=2). In the Schulz et al. and the unpublished dataset, no collected data were excluded. For the joint analysis of the different datasets, we filtered the dataset from Meder et al. and Schulz et al. to only use participants assigned to one of the experimental conditions (smooth environments). In doing so, we removed 50 participants from Meder et al. and 81 participants from Schulz et al.
Non-participation	We had no non-participants.
Randomization	Participants were not allocated into experimental groups.

Reporting for specific materials, systems and methods

We require information from authors about some types of materials, experimental systems and methods used in many studies. Here, indicate whether each material, system or method listed is relevant to your study. If you are not sure if a list item applies to your research, read the appropriate section before selecting a response.

Materials & experimental systems

n/a	Involved in the study
☒	☐ Antibodies
☒	☐ Eukaryotic cell lines
☒	☐ Palaeontology and archaeology
☒	☐ Animals and other organisms
☒	☐ Clinical data
☒	☐ Dual use research of concern
☒	☐ Plants

Methods

n/a	Involved in the study
☒	☐ ChIP-seq
☒	☐ Flow cytometry
☒	☐ MRI-based neuroimaging
